# Blood Metal Ion Release After Primary Total Knee Arthroplasty: A Prospective Study

**DOI:** 10.1111/os.12591

**Published:** 2020-02-05

**Authors:** Tobias Reiner, Reza Sorbi, Maike Müller, Timo Nees, Jan Philippe Kretzer, Markus Rickert, Babak Moradi

**Affiliations:** ^1^ Center for Orthopedics, Trauma Surgery and Spinal Cord Injury Heidelberg University Hospital Heidelberg Germany; ^2^ Laboratory of Biomechanics and Implant Research, Center for Orthopedics, Trauma Surgery and Spinal Cord Injury Heidelberg University Hospital Heidelberg Germany; ^3^ Department of Orthopaedics and Orthopaedic Surgery University Hospital Giessen and Marburg (UKGM) Giessen Germany

**Keywords:** Metal ions, Metal wear, Total knee arthroplasty (TKA)

## Abstract

**Objectives:**

To investigate the course of *in vivo* blood metal ion levels in patients undergoing primary total knee arthroplasty (TKA) and to investigate potential risk factors associated with metal ion release in these patients.

**Methods:**

Twenty‐five patients with indication for TKA were included in this prospective study. Whole blood metal ion analysis was performed pre‐operatively and at 1 week, 6 weeks, 3 months, 6 months, and 12 months postoperatively. Clinical scores were obtained using the American Knee Society Score (AKSS) and the Oxford Knee Score (OKS) at each follow‐up and patientsʼ activity levels were assessed by measuring the mean annual walking cycles at 12 months follow‐up. Anteroposterior and lateral radiographs of the operated knee were evaluated postoperatively and at 12‐month follow‐up with regard to implant position and radiological signs of implant loosening. Correlation analysis using multivariate linear regression was performed to investigate the influence of different variables (age, gender, functional scores, number of walking cycles, and body mass index [BMI]) on blood cobalt ion concentrations.

**Results:**

Mean metal ion levels of cobalt, chromium, molybdenum, and titanium were 0.28 μg/L (SD, 0.14), 0.43 μg/L (SD, 0.49), 0.62 μg/L (SD, 0.45), and 1.96 μg/L (SD, 0.98), respectively at 12‐month follow‐up. Mean cobalt ion levels significantly increased 1‐year after surgery compared to preoperative measurements. There was no statistically significant increase of mean metal ion levels of chromium, titanium, and molybdenum at 1‐year follow‐up. Overall, metal ion levels were low and no patient demonstrated cobalt ion levels above 1 μg/L. Postoperative radiographs demonstrated well‐aligned TKAs in all patients and no signs of osteolysis or implant loosening were detected at 1‐year follow‐up. Both the AKSS and OKS significantly improved during the course of the study up to the final follow‐up. Multivariate regression analysis did not show a statistically significant correlation between the tested variables and blood cobalt ion concentrations.

**Conclusion:**

A statistically significant increase of mean cobalt ion concentration at 1‐year follow‐up was found in this cohort of patients with well‐functioning TKA, although overall blood metal ion levels were relatively low. Despite low systemic metal ion concentrations seen in this cohort, the local effects of increased metal ion concentrations in the periprosthetic environment on the long‐term outcome of TKA should be further investigated.

## Introduction

Total knee arthroplasty (TKA) is a successful treatment option for advanced osteoarthritis of the knee with reported long‐term survival rates of 91% after 20 years^1^. Aseptic component loosening remains the main reason for revision surgery in the long term which accounts for up to 32% of all TKA revisions according to registry data^2^. Although the pathomechanisms of implant failure are multifactorial, wear‐related complications play a major role in the development of periprosthetic osteolysis and late implant loosening.

Periprosthetic osteolysis can be considered a consequence of either increased bone resorption or decreased bone formation around the orthopedic implant. Accumulating metal wear particles and metal ions are able to stimulate both bone metabolism and the immune system through different pathways hereby contributing to the pathomechanisms of implant loosening^3^. As part of a foreign body reaction, the role of macrophages and their activation through phagocytosis of wear particles is well understood^4^, ^5^, ^6^. However, less is known about the effects of accumulating metal ions on periprosthetic bone metabolism and their interactions with the immune system. With regard to bone metabolism, metal ions are able to recruit and activate osteoclast precursor cells and have the potential to inhibit osteoblasts at the same time^7^, ^8^, ^9^. Both mechanisms play an important role in the development of periprosthetic osteolysis. For example, Cadosch *et al*. and others could show that metal ions are able to induce the expression of different chemotactic cytokines in macrophages and osteoclasts, such as CCL17/TARC (thymus and activation‐regulated chemokine) and CCL22/MDC (macrophage‐derived chemokine), that are involved in the recruitment of osteoclast precursor cells which might ultimately stimulate periprosthetic osteolysis and implant loosening^7^, ^10^, ^11^, ^12^, ^13^, ^14^. Other studies have demonstrated that nontoxic concentrations of metal ions are able to suppress the differentiation and function of osteoblastic cells *in vitro* which could contribute to implant loosening by inhibition of periprosthetic bone formation^8^, ^9^.

Due to their size, metal ions are able to pass into the systemic blood circulation where they form complexes with serum proteins^15^. These hapten‐like metal‐protein‐complexes can be recognized by T‐lymphocytes as an antigen, which leads to their proliferation and the production of chemotactic cytokines, such as interleukin (IL)‐6 and tumor necrosis factor‐alpha (TNF‐a) that might, in turn, trigger a specific immune response and activate osteoclastogenesis^3^, ^16^, ^17^, ^18^. In this context, delayed type IV hypersensitivity reactions to metal ions have become of increasing interest, and orthopaedic surgeons are facing a growing demand for coated, so‐called hypoallergenic total knee prosthesis^19^, ^20^, ^21^.

In joint replacements, metal ions are generated by corrosive degradation of metal wear debris and by tribocorrosion at the exposed metal surfaces of the implant. *In vivo*, a passive oxide film is spontaneously formed on the metallic surface which protects the implant material from corrosion and inhibits the release of metal ions^22^. However, repetitive mechanical stress leads to the disruption of this protective oxide film hereby facilitating corrosion damage and metal ion release^23^. In addition, galvanic corrosion can occur at taper connections of modular implants, when mixed metal components with different electrochemical potentials are combined^24^, ^25^. The two dissimilar metals then act as anode and cathode in the presence of an electrolyte, which facilitates metal ion generation^26^. Besides these aforementioned electrochemical corrosive mechanisms, *in vitro* studies could demonstrate that osteoclasts are able to directly corrode metallic biomaterials and release metal ions by leaving resorption pits on the metal surface^3^, ^27^.

Modular junctions and large metal surface areas, which are prone to fretting damage and corrosion, are both commonly found in TKAs, which might lead to the generation and accumulation of metal ions in total knee replacements^28^. Although simulator studies have shown that relevant amounts of metal wear products and metal ions are continuously released from total knee prosthesis^29^, little is known about *in vivo* metal ion levels in patients following well‐functioning primary total knee replacement.

Therefore, the aim of this prospective study was: (i) to investigate *in vivo* blood metal ion concentrations of cobalt, chromium, molybdenum, and titanium over time in patients undergoing primary total knee replacement; (ii) to assess clinical and radiological outcome; and (iii) to investigate the influence of potential risk factors on blood metal ion concentrations in these patients.

## Materials and Methods

### *Inclusion and Exclusion Criteria*

The inclusion criteria were: (i) patients aged between 35 and 85 years; and (ii) advanced primary or secondary osteoarthritis of the knee requiring TKA. Exclusion criteria were: (i) patients with existing metal implants or occupational exposure to toxic metals in order to rule out additional sources of metal ion exposure; (ii) patients with a history of hypersensitivity reaction to metals; and (iii) patients with severe renal insufficiency or any other medical condition that would impair their participation in this study.

### *Patients*

**Figure 1:**
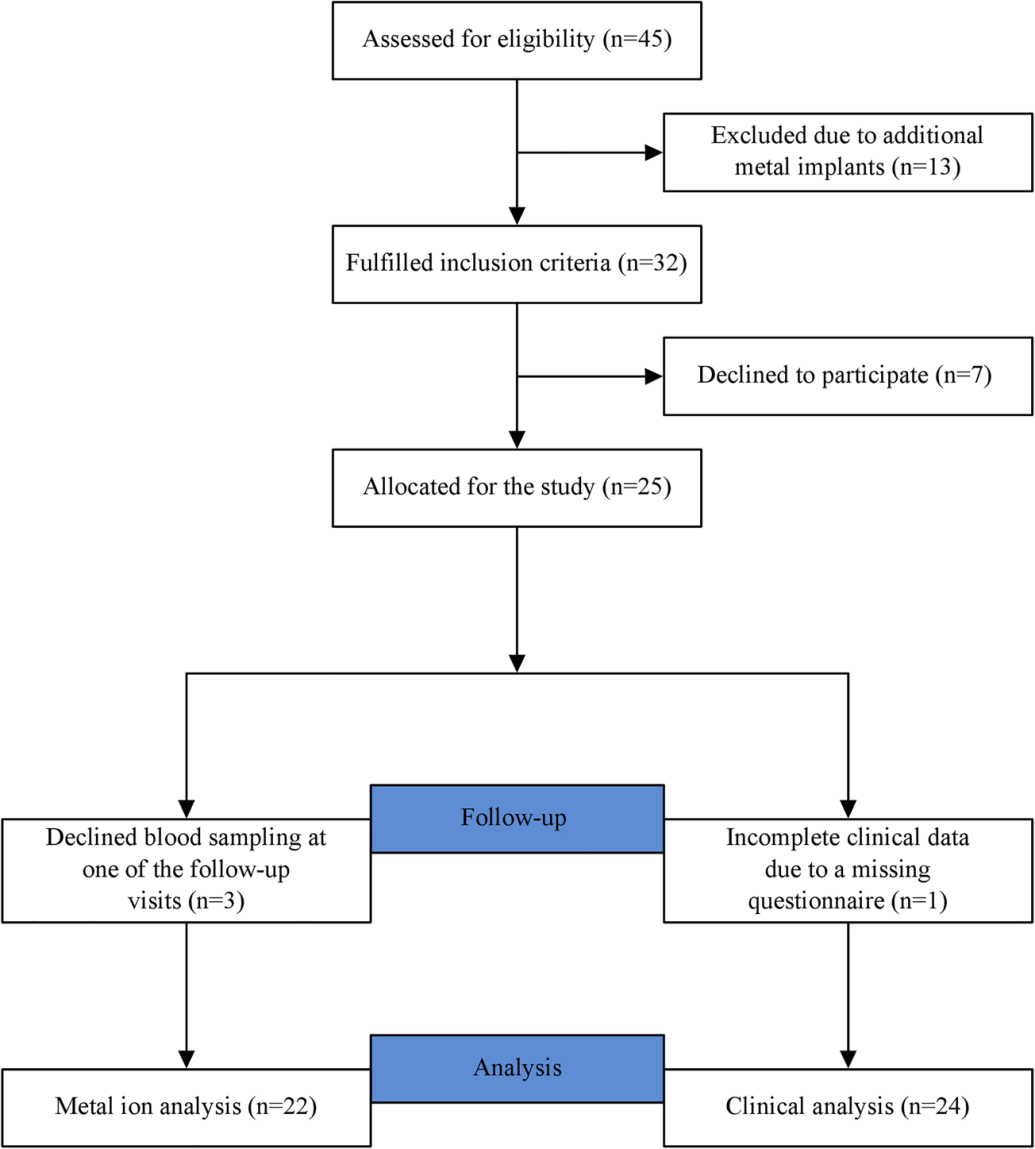
The flowchart illustrates patient enrolment.

Between March 2010 and December 2010, we included 25 patients who were allocated for primary TKA at our institution in this prospective single‐center study. Patients were recruited of a consecutive cohort of 32 patients who were allocated for primary TKA at our institution and who fulfilled the inclusion criteria. Figure 1 illustrates patient enrolment. Seven patients denied participation in the study before study enrolment. From the remaining 25 patients, three patients had to be excluded from metal ion analysis due to incomplete data as they denied blood sampling at one of the follow‐up visits. One patient had to be excluded from clinical analysis due to a missing questionnaire. The study was approved by the local ethics committee (No S‐056/2010) and performed in accordance with the Helsinki Declaration. Written informed consent was obtained by every patient before study inclusion.

### *Surgery*

TKA was performed with a cemented total knee replacement (P.F.C. ® SIGMA® Total Knee System, DePuy Orthopedics Inc., Warsaw, USA). A posterior cruciate ligament retaining femoral component was used in 19 patients and a cruciate substituting femoral component was used in four patients. A cemented all‐polyethylene resurfacing of the patella was performed in six patients. A fixed bearing, highly cross‐linked polyethylene insert was used in all patients. In two patients an intra‐operative decision was made to use a semi‐constrained total knee replacement (P.F.C. ® SIGMA® TC3 Knee System DePuy Orthopedics Inc., Warsaw, USA) due to insufficiency of the lateral collateral ligament. The femoral components were made of cast CoCr28Mo6 alloy and the tibial components consisted of wrought Ti6Al4V alloy. TKA was performed according to the manufacturer's instructions using a medial parapatellar approach.

### *Outcome Measures*

#### *Metal Ion Measurements*

The primary outcome measures were whole blood metal ion concentrations of cobalt, chromium, molybdenum, and titanium that were measured at each follow‐up interval. Whole blood metal ion analysis was performed preoperatively and at 1 week, 6 weeks, 3 months, 6 months, and 12 months postoperatively. Metal ion concentrations were measured using high‐resolution inductively coupled plasma‐mass spectrometry (HR‐ICP‐MS, Element 2, Thermo Fisher Scientific, Bremen, Germany). Blood samples were collected using a blood collection system intended for trace metal ion analysis (Sarstedt, Nuembrecht, Germany; Refs. 58.1162.600 and 01.1604.400). All blood samples were stored at −20°C and analyzed at the same time in order to minimize calibration errors of the spectrometer. Before analysis, the samples were digested with high‐purity nitric acid (HNO_3_) and hydrogen peroxide under clean‐room conditions in a high‐pressure microwave autoclave (UltraClave II, Milestone, Bergamo, Italy)^30^, ^31^. Metal ion measurements were repeated three times in every sample and mean values of metal ion concentrations for each sample were calculated. Detection limits of 0.005 μg/L for cobalt, 0.02 μg/L for chromium, 0.01 μg/L for molybdenum, and 0.06 μg/L for titanium have been established for this method^30^. In addition, serum creatinine values were measured for each patient in order to rule out severe renal insufficiency.

#### *American Knee Society Score (AKSS) and the Oxford Knee Score (OKS)*

Secondary outcome parameters were the clinical and functional outcome after TKA that were assessed using patient reported outcome measures. The clinical and the functional AKSS and the OKS were evaluated at each follow‐up. Both the clinical and the functional AKSS are ranging from 0 (worst) to 100 (best) while the OKS is ranging from 0 (worst) to 48 (best)^32^, ^33^.

#### *Patient Activity Levels*

Patients’ estimated annual walking cycles were assessed with a StepWatch™ Activity Monitor (Orthocare Innovations LLC, Edmonds, WA, USA) 1‐year after surgery by measuring the number of walking cycles per day over a period of 2 weeks. The estimated annual walking cycles were obtained by multiplying the measured mean daily walking cycles for each patient by 365.

#### *Radiological Outcome Measures*

Anteroposterior and lateral radiographs of the operated knee were evaluated postoperatively and at 12‐month follow‐up with regard to implant alignment and signs of periprosthetic radiolucent lines and osteolysis. Mean postoperative femorotibial angle and mean varus/valgus orientation of the tibial component were measured on anteroposterior radiographs of the knee.

### *Statistical Methods*

Statistical analysis was performed using the software SPSS® (version 23.0; SPSS IBM Corp., Chicago, IL, USA). Data were evaluated descriptively as arithmetic mean, standard deviation, minimum, and maximum. Analysis of variance for repeated measures (ANOVA) with *post hoc* Bonferroni‐correction for multiple comparisons was performed, in order to compare the differences in mean metal ion concentrations and clinical scores at each follow‐up. Correlation between blood cobalt ion levels and clinical variables (age, gender, AKSS, OKS, body mass index [BMI], and number of walking cycles) was assessed using the Spearman rank correlation coefficient and multiple linear regression analysis. All tests were two‐sided and a *P*‐value < 0.05 was considered significant.

## Results

### *General Results*

A total of 25 patients (13 male and 12 female patients) were included in the present study. The mean age of the patients at time of surgery was 64.7 years (range, 42–81 years). Indication for TKA was primary osteoarthritis in 22 patients (88%) and post‐traumatic osteoarthritis in three patients (12%). Mean BMI before surgery was 31.6 kg/m^2^ (range, 19–46 kg/m^2^). Mean creatinine values were 0.99 mg/dl (range, 0.8–1.6 mg/dl). No patient of the study cohort had to be excluded due to severe renal insufficiency.

### *Results of Metal Ion Analysis*

Results of metal ion analysis are summarized in Table 1. We found no statistically significant difference in mean metal ion concentrations of chromium, titanium, and molybdenum at 1‐year follow‐up compared to preoperative metal ion levels. Mean cobalt ion concentrations significantly increased at all follow‐up intervals compared to preoperative measurements, with a slight decrease between the 3‐month and 6‐month follow‐ups. In order to rule out the influence of a higher constrained implant design (PS and TC3) on metal ion release, a subgroup analysis of the cruciate‐retaining implants (n = 17) was performed, which demonstrated similar results regarding the statistically significant differences of cobalt ion concentrations at 1‐year follow‐up compared to preoperative measurements (*P* < 0.001). No statistically significant increase of chromium (*P* = 1.00), molybdenum (*P* = 1.00), and titanium ion concentrations (*P* = 0.295) was found in the subgroup analysis at 1‐year follow‐up.

**Table 1:** Metal ion measurements in μg/L at the different follow‐up intervals (n = 22)

	Pre‐operation (T0)	6 weeks post‐op (T1)	3 months post‐op (T2)	6 months post‐op (T3)	1 year post‐op (T4)	*P*‐value			
						T0 vs T1	T0 vs T2	T0 vs T3	T0 vs T4
Cobalt	0.023 ± 0.04	0.110 ± 0.20	0.172 ± 0.16	0.060 ± 0.07	0.279 ± 0.14	**0.035**	**<0.001**	**0.026**	**<0.001**
	0.006 (0.005–0.141)	0.037 (0.005–0.896)	0.105 (0.006–0.603)	0.025 (0.005–0.229)	0.243 (0.122–0.615)				
Chromium	0.318 ± 0.28	0.255 ± 0.18	0.123 ± 0.15	0.167 ± 0.12	0.430 ± 0.49	0.384	**0.008**	**0.024**	0.353
	0.251 (0.052–1.297)	0.206 (0.022–0.671)	0.069 (0.005–0.469)	0.126 (0.056–0.486)	0.268 (0.000–1.984)				
Molybdenum	0.489 ± 0.26	0.545 ± 0.27	0.555 ± 0.59	0.451 ± 0.27	0.622 ± 0.45	0.327	0.534	0.598	0.190
	0.421 (0.225–1.221)	0.489 (0.244–1.479)	0.404 (0.168–2.967)	0.355 (0.174–1.164)	0.456 (0.212–1.827)				
Titanium	1.417 ± 1.37	1.831 ± 0.84	2.346 ± 1.03	1.177 ± 0.80	1.960 ± 0.98	0.244	**0.020**	0.549	0.133
	1.077 (0.006–4.517)	1.680 (0.797–3.142)	2.446 (0.006–3.752)	1.137 (0.006–4.058)	1.837 (0.598–4.28)				

Note: Data were expressed as mean, SD (upper row) and median, range (lower row). Analysis of variance for repeated measures (ANOVA) with *post hoc* Bonferroni‐correction was used for comparison between the intervals. A *P*‐value < 0.05 was considered statistically significant.

No patient demonstrated cobalt ion levels higher than 1μg/L. Chromium ion levels significantly decreased between the 3‐month and 6‐month follow‐ups compared to preoperative measurements. Two patients showed chromium ion levels above 1μg/L at the 1‐year follow‐up visit. In these patients, there was no evidence of component malposition or signs of mechanical failure on plain radiographs.

### *Outcome of AKSS and the OKS*

Both the AKSS and OKS significantly improved during the course of the study up to the final follow‐up (Table 2).

**Table 2:** American Knee Society score (AKSS) and the Oxford Knee score (OKS) as measured at the different follow‐up intervals (n = 24)

Mean ± SD (Range)	Pre‐operation (T0)	6 weeks post‐op (T1)	3 months post‐op (T2)	6 months post‐op (T3)	1 year post‐op (T4)	*P*‐value			
						T0 vs T1	T0 vs T2	T0 vs T3	T0 vs T4
Clinical (AKSS)	32.5 ± 11.8 (13–55)	70.6 ± 18.9 (32–92)	78.3 ± 15 (37–99)	82.6 ± 15.1 (28–95)	84.8 ± 13.5 (30–100)	**<0.001**	**<0.001**	**<0.001**	**<0.001**
Functional (AKSS)	41.7 ± 14.2 (5–80)	45.8 ± 19.7 (15–90)	64.8 ± 14.5 (35–90)	74.4 ± 20.9 (15–100)	78.6 ± 21.1 (15–100)	0.394	**<0.001**	**<0.001**	**<0.001**
Oxford knee Score (OKS)	18.9 ± 6.9 (9–34)	31.3 ± 9 (7–44)	33 ± 8.7 (7–44)	39.1 ± 8 (6–48)	39.7 ± 8.5 (5–40)	**<0.001**	**<0.001**	**<0.001**	**<0.001**

Note: Data were expressed as mean, standard deviation (SD) and range. Analysis of variance for repeated measures (ANOVA) with *post hoc* Bonferroni‐correction was used for comparison between the intervals. A *P*‐value < 0.05 was considered statistically significant.

### *Outcome of Patients Activity Levels*

Mean annual walking cycles were 1,566,219 (range, 776,776–2,484,920). Multivariate regression analysis showed no correlation between blood cobalt ion concentrations at 12‐months follow‐up and gender (Beta = −0.17, *P* = 0.552), age at time of surgery (Beta = −0.17, *P* = 0.613), BMI (Beta = −0.38, *P* = 0.341), number of walking cycles (Beta = 0.08, *P* = 0.788), or OKS (Beta = −0.45, *P* = 0.427).

### *Radiological Outcome*

**Figure 2:**
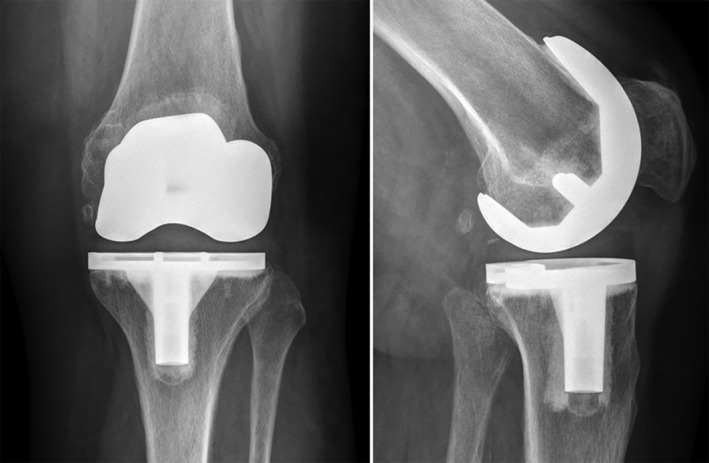
The radiograph shows the x‐ray images of the left knee of a 72‐year‐old female patient at 1‐year follow‐up demonstrating a well‐aligned implant position.

Postoperative radiographs demonstrated well‐aligned TKAs in all patients. Mean postoperative femorotibial angle was 176.4 degrees (range, 174–179; SD, 1.5 degrees). Mean varus/valgus orientation of the tibial component was 90.2 degrees (range, 89–93; SD, 0.97 degrees). No signs of osteolysis or implant loosening were detected at 1‐year follow‐up (Fig. 2).

## Discussion

Blood metal ion analysis has proved to be a viable tool in the follow‐up of patients with metal‐on‐metal total hip arthroplasties in order to detect excessive metal ion release associated with increased wear or corrosion. In TKA, wear related problems have been typically reported with regard to damage at the polyethylene insert. Although retrieval studies have recently demonstrated that abrasive and corrosive damages commonly occur at the femoral component of total knee replacements, little is known about the metal ion exposure in patients with well‐functioning TKA^34^. Therefore, the aim of this prospective study was to determine *in vivo* blood metal ion levels in patients following primary TKA and to investigate potential risk factors for increased metal ion release in these patients.

We found no statistically significant difference in mean metal ion concentrations of chromium, titanium, and molybdenum at 1‐year follow‐up compared to preoperative metal ion levels. Mean cobalt ion concentration significantly increased 1‐year after surgery. However, absolute cobalt ion concentrations were low and no patient demonstrated cobalt ion levels higher than 1 μg/L at the 1‐year follow‐up visit. This is in contrast to the findings of a cross‐sectional study in which elevated metal ion levels were found 5–7 years after primary total knee replacement with median chromium ion concentrations of 0.92 μg/L and cobalt ion concentrations of 3.28 μg/L.^35^ Postler *et al*. reported elevated chromium levels 1‐year after TKA with 11% of patients in this cohort demonstrating chromium ion concentrations above 2 μg/L, which is considered to be a potentially critical value for metal‐on‐metal hip arthroplasties according to current guidelines^36^, ^37^. In another study by Lons *et al*., a significant increase in blood metal ion concentrations of cobalt, chromium, and titanium was found 1‐year after primary TKA with mean ion levels of 1.27 μg/L, 1.41 μg/L, and 4.08 μg/L, respectively^38^. We could not confirm these findings with mean cobalt, chromium, and titanium levels of 0.28 μg/L, 0.43 μg/L, and 1.96 μg/L at 1‐year follow‐up. Clinical results demonstrated significant functional improvement at short‐term follow‐up in our study, which were comparable to those reported in the literature^2^. No influence of clinical scores, BMI, or patient activity levels on blood metal ion concentrations was observed, which is in accordance to the findings of other studies investigating blood metal ion release of total knee replacements^35^, ^38^.

Although wear in TKA primarily consists of polyethylene debris, metal ions and particles are potentially released by well‐functioning TKAs. A retrieval analysis of 52 total knee replacements demonstrated that metal loss at the articulating surface of the femoral components caused by scratching damage and corrosion was present in 98% of the retrievals^34^. Kretzer *et al*. investigated polyethylene and metal wear products released by TKAs in a simulator study and found that 12% by weight of the wear was metallic^29^. The main mechanisms of metal ion generation in TKA are mechanical wear and corrosion. Abrasive damage at the articulating surface of the femoral component leads to the generation of metal debris, and the electrochemical degradation of these wear particles ultimately results in the formation of metal ions. In addition, metal ions can be released from the implant bulk by different corrosion mechanisms such as inflammatory cell‐induced corrosion (ICIC) or mechanically assisted crevice corrosion (MACC) at modular taper junctions^39^, ^40^. The clinical effects of accumulating metal wear products and metal ions in the periprosthetic environment of total knee replacements are still not completely understood. Metal ions are able to influence both the immune system and bone metabolism, which might result in periprosthetic osteolysis, implant loosening, or adverse local tissue reactions^41^. Reports of pseudotumor formation after total knee replacements are rare and have been usually associated with extensive metal wear due to full‐thickness damage of the polyethylene insert or taper corrosion of modular total knee megaprostheses^42^, ^43^, ^44^.

There are limitations to the present study that have to be mentioned. The relatively small sample size of 25 patients has to be considered as the main limitation of the study. This was mainly attributed to the exclusion criterion regarding additional metal implants or history of metal hypersensitivity reactions, which led to the exclusion of patients during the recruitment period. However, due to the longitudinal study design and the high standardization and accuracy of the methodology used for metal ion analysis, we consider our results to be representative for larger patient cohorts. In addition, the follow‐up period was short and a longer follow‐up duration may influence metal ion concentrations. However, other studies demonstrated that metal ion levels are supposed to be consistent over time in patients with well‐functioning TKA^38^, ^45^, ^46^, and simulator studies have shown that the kinetics of metal ion release of TKA seem to differ from those of metal‐on‐metal total hip arthroplasties^29^, in which the highest wear rates occur during the running‐in phase in the first postoperative year followed by a decreased steady‐state wear afterwards. In contrast, metal‐on‐polyethylene articulations in well‐functioning TKA are supposed to steadily release metal wear products without alteration in wear progression, which is mainly attributed to a distinct mode of lubrications and the viscoelasticity of the polyethylene, when compared to metal‐on‐metal articulations^29^. Therefore, we believe that the 1‐year follow‐up of this study design is representative for longer follow‐up periods, although it would be interesting to investigate the kinetics of metal ion release of well‐functioning TKA with longer follow‐up intervals in future studies.

### *Conclusion*

In conclusion, we found that cobalt ion levels significantly increased 1‐year after surgery compared to preoperative measurements in this cohort of patients with well‐functioning primary total knee replacements. However, absolute metal ion levels were relatively low and no patient demonstrated cobalt ion levels above 1 μg/L. Despite low systemic metal ion concentrations seen in this cohort, the accumulation of metal ions in the periprosthetic environment could play a role in the development of hypersensitivity reactions or implant loosening, and the local effects of increased metal ion concentrations on the long‐term outcome of TKA should be further investigated.
